# Development of curcumin‐loaded *Prunus armeniaca* gum nanoparticles: Synthesis, characterization, control release behavior, and evaluation of anticancer and antimicrobial properties

**DOI:** 10.1002/fsn3.2562

**Published:** 2021-09-08

**Authors:** Davoud Salarbashi, Mohsen Tafaghodi, Morteza Fathi, Seyyed Mohammad aboutorabzade, Farzaneh Sabbagh

**Affiliations:** ^1^ Nanomedicine Research Center School of Medicine Gonabad University of Medical Sciences Gonabad Iran; ^2^ Department of food science and nutrition School of Medicine Gonabad University of Medical Sciences Gonabad Iran; ^3^ Nanotechnology Research Center Pharmaceutical Technology Institute Mashhad University of Medical Sciences Mashhad Iran; ^4^ Pharmaceutics Department School of Pharmacy Mashhad University of Medical Sciences Mashhad Iran; ^5^ Health Research Center Life Style Institute Baqiyatallah University of Medical Sciences Tehran Iran; ^6^ Department of Medicinal Chemistry School of Pharmacy Mashhad University of Medical Sciences Mashhad Iran; ^7^ Department of Chemical Engineering Chungbuk National University Cheongju Korea

**Keywords:** curcumin, encapsulation, nanoparticles, *Prunus armeniaca* gum exudates, release study

## Abstract

The present work was conducted to develop a new polysaccharide‐based encapsulation system via electrostatic interactions between *Prunus armeniaca* gum exudates (PAGE) and Ca^2+^ ions to enhance the biological activity and bioavailability of curcumin. The effects of different levels of pH (6, 7, and 8) and ion concentrations (1, 3, and 5) on the particle diameter and surface charge of the samples were examined. The encapsulation efficiency in the PAGE‐based nanoparticles was realized to be 86.1%, indicating the encapsulation technique applied in this study was effective to entrap most of the curcumin within the PAGE matrix. The nanoparticles showed a smooth surface with spherical shape. Fourier transform infrared spectroscopy (FT‐IR) and X‐ray diffraction (X‐ray) studies confirmed the formation of polyelectrolyte complexation. The cumulative release of curcumin in simulated gastrointestinal tract was less than 75%, revealing a gradual release trend. Both pure curcumin and curcumin‐loaded nanoparticles were toxic to the cancer cell lines.

## Graphical abstract

Encapsulation of curcumin into PAGE‐based NPs improved the anticancer activity of curcumin. Curcumin‐loaded PAGE‐based NPs showed greater antibacterial activity than free curcumin.

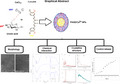

## 1 INTRODUCTION

Curcumin has anti‐HIV, antioxidant, anticancer, antimicrobial, and anti‐inflammatory properties (Adamczak et al., 2020; Aliabbasi et al., 2021; Han & Yang, 2005; Solghi et al., 2020; Zorofchian Moghadamtousi et al., 2014). However, the poor water solubility of curcumin causes significant challenges in producing formulations with sufficiently high bio‐accessibility (Gera et al., 2017). Different studies have been performed to enhance water solubility and reduce this bioactive agent's administration dose (Basniwal et al., 2011; Kim et al., 2011; Kurien et al., 2007; Liu et al., 2020). The entrapment of curcumin into the polymeric matrices is known as one of the most promising approaches to overcome this issue.

Nano‐encapsulation is used to incorporate sensitive ingredients into nanoscale particles, which can preserve the core against surrounding conditions. Furthermore, it uses to target delivery and controllable release of ingredients (Chatzikleanthous et al., 2020; Seyedabadi et al., 2021). Since the coacervation method has various advantages such as the high loading capacity, low‐temperature operation, and more competency in controlling the release rate of ingredients, the use of this technique to encapsulate phytochemicals has attracted the attention of scientists in recent years (Taneja & Singh, 2012). The wall material used for encapsulation should be at least nontoxic and biocompatible. Polysaccharide‐based encapsulant materials are broadly used for this purpose due to their advantages such as nontoxicity, biodegradability, and biocompatibility (Elzoghby et al., 2012).

*Prunus armeniaca L* is the most frequently cultivated apricot species, globally distributed in China, the Himalaya region, Iran, Pakistan, and other parts of temperate Asia. Its polysaccharide exudates have been utilized as medicinal remedies in Iran (Fathi et al., 2016). To date, various studies have been carried out to encapsulate curcumin into polysaccharide‐based encapsulation systems. In a study conducted by Goelo et al. (2020), four encapsulating agents including pectin, maltodextrin, inulin, and xanthan were used to develop curcumin‐loaded encapsulation systems. A product yield between 7.52% and 60.60% was reported for all the developed encapsulation vehicles. The authors found that Weibull model could effectively describe the release data. In another work, the capability of different polysaccharides including levan, fucoidan, alginate, guar gum, and κ‐carrageenan for encapsulation of curcumin were examined (Richa & Choudhury, 2020). It was observed that the highest encapsulation efficiency was obtained when κ‐carrageenan was used as encapsulating agent. Iurciuc‐Tincu et al. (2020) incorporated curcumin into several polysaccharide‐based microstructural vehicles (gellan, i‐carrageenan, and chitosan) to increase its stability and bioavailability. The encapsulation efficiency was in the range of 85.75%–97.25%, exhibiting the developed encapsulation systems were effective to entrap most of the curcumin within the polysaccharides matrices.

In the present paper, for the first time, PAGE was applied to encapsulate curcumin, as a biological compound. The structure and crystallinity of curcumin after encapsulation were evaluated. Furthermore, the effect of encapsulation on antimicrobial and anticancer activities of curcumin was tested. Besides, the morphology of nanocapsules was investigated by scanning electron microscopy (SEM). The release of curcumin from the developed system was investigated at the simulated circumstances to explore its potential utilization in different industries. Finally, the effect of PAGE, PAGE and curcumin, and curcumin‐loaded PAGE nanoparticles dispersions as edible coating was examined on the shelf life of shrimp during storage period.

## 2 MATERIALS AND METHODS

### 2.1 Materials

Curcumin was supplied from Sigma Aldrich Co (˃96%, stored at −20℃). Other chemicals used in the present research were of analytical grade and purchased from Merck. Microbial cultures were obtained from Himedia. PAGE was collected from *Prunus armeniaca* trees in Bojnord, Rooin during spring (37 12 min 13.9 s North and 57 29 min 13.5 s East). The purification of the gum was performed as described by Fathi et al. (2017). Fresh shrimp was purchased from the local market in Tehran, Iran.

### 2.2 Methods

#### 2.2.1 Fabrication of nanoparticles

To prepare the PAGE solution, the PAGE powder was dissolved in deionized water with different pH levels (6, 7, and 8) and then kept overnight to be hydrated completely. The ethanolic solution of curcumin (400 μl, 10 mg/ml) was added to 1 ml polymeric solution. Afterward, calcium chloride solution was injected into the mixture with a mixture ratio of 1:1 under vortexing with a flow rate of 1 ml/min at ambient temperature (23 ± 2°C) (Naji‐Tabasi et al., 2017). The excess values of CaCl_2_ were removed by centrifugation (Kubota, Tokyo, Japan) at 3000 *g* for 5 min. Finally, the ethanol was evaporated by a rotary evaporator (HB4basic, IKA, Germany) and the nanoparticles dispersion was kept at 4°C until further analysis.

#### 2.2.2 Particle diameter and size distribution analysis

The nanoparticle diameter was measured using a PALS‐ Malvern Zetasizer Nano ZS (Malvern Instruments Ltd., Worcestershire, UK) at a fixed scattering angle of 90° (25 ± 0.1℃). To avoid multiple scattering phenomena, the nanoparticle's solutions were diluted (Tan et al., 2013).

#### 2.2.3 Zeta potential (*ξ*) measurement

The ***ξ*** values of the nanoparticles were measured using a Malvern Nanosizer (model Nano‐S). *ξ* values of the nanoparticles were determined under holder temperature of 25℃ and electrical voltage 3.9 V.

#### 2.2.4 Scanning electron microscopy (SEM)

The morphological properties of the nanoparticles were examined using scanning electron microscopy (SEM) (MIRA3 TESCAN). For SEM characterization, a drop of nanoparticles solution was air‐dried on a slide, fixed with double‐sided sticky tape, and then coated by a sputter coater for 40 s.

#### 2.2.5 Fourier transform infrared (FT‐IR) spectroscopy

The FT‐IR analysis (AVATAR 370 FT‐IR, Thermo Nicolet) was conducted to evaluate the chemical structures of PAGE, CaCl_2_, and PAGE nanoparticles. The nanoparticles were freeze‐dried to get nanoparticle powder. Then, 2 mg of nanoparticles was grounded and pressed into a pellet with potassium bromide (KBr) powder. The scanning was carried out in the wavenumber range of 400–4000 cm^−1^.

#### 2.2.6 XRD measurements

The XRD analysis of PAGE, CaCl_2_, and nanoparticles was carried out by an X‐ray diffractometer (Bruker, Germany), with a range of 10°–90°.

#### 2.2.7 Encapsulation efficiency (EE) analysis

1 ml encapsulated curcumin was centrifuged (12,000 *g*, 20 min), and the supernatant was separated. Acetone was added to the supernatant with a ratio of 9:1. Afterward, the absorbance was read at 426 nm by a UV‐vis spectrophotometer (a Lambda 25‐Perkin Elmer, Waltham, MA, USA; Zou et al., 2016). EE was quantified based on the following equation:

(1) $$\text{EE}\left(\%\right)=\frac{\text{Encapsulated curcumin}\left(\text{mg}\right)}{\text{The total curcumin added}\left(\text{mg}\right)}\times 100$$

#### 2.2.8 Release studies of curcumin

The release profile of curcumin in simulated gastrointestinal tract was tested.

##### Simulated Gastric Fluid (SGF) preparation

To prepare SGF, first, 0.2 g of NaCl and 0.32 g pepsin were dissolved in a 100 ml deionized water. Afterward, pH of the solution was adjusted to 1.8 using 0.1 N HCl. The resulting solution was then stirred at a constant temperature of 37ᵒC.

##### Simulated Intestinal Fluid (SIF) preparation

To prepare SIF, 0.68 g buffer phosphate was added to 25 ml distilled water. In the next step, 77 ml of 0.2 M NaOH and 50 ml of water were added to the resulting solution. Pancreatin (1 g) was added to the solution, followed by adjusting pH to 6.5. The volume made up with distilled water to 100 ml and kept at 37°C (Abazović et al., 2006).

#### 2.2.9 Release kinetics

To describe the release kinetics of curcumin from PAGE nanoparticles, various models, including zero‐order (Equation 2), First‐order (Equation 3) Higuchi (Equation 4), and Peppas (Equation (5)), were used (Higuchi, 1963, Korsmeyer et al., 1983):

Zero order model:

(2) $$M_{0}‐M_{t}=k_{0}\cdot t$$

First‐order model:

(3) $$\text{Ln}\left(M_{0}/M_{t}\right)=k\cdot t$$

here, *M_t_*/*M*_∞_ represents the fraction of curcumin released at time t.

Higuchi model:

(4) $$Q=k_{H}t^{1/2}$$

in which, *k_H_* is known as the Higuchi dissolution constant.

Peppas model:

(5) $$M_{t}/M_{\infty }=k_{P}t^{n}$$

In this equation, *M_t_*/*M*_∞_ represents the fraction of curcumin released at time *t, n* shows diffusion and exponent, and *kp* is kinetic constant.

#### 2.2.10 Antibacterial capacity

The antibacterial capacity of pure curcumin and curcumin‐loaded PAGE NPs was examined against *E. coli*, a gram‐negative bacteria and *S. aureus*, as a gram‐positive bacteria. The disk diffusion technique was employed to examine the antibacterial capacity of the free and encapsulated curcumin.

#### 2.2.11 In vitro cytotoxicity

MTT technique was employed to evaluate the cytotoxicity of the pure and encapsulated curcumin. For this purpose, two cell lines including 4t1 (a breast cancer cell line) and A2780 (a human ovarian cancer cell line) were used. All the tested cells were seeded into 96‐well plates, and then, the plates were incubated at 37℃ for 24 h. Afterward, pure and encapsulated curcumin were incorporated into plates and plates were again incubated for 24 h. In the next step, a medium comprising MMT reagent was replaced with the previous one and incubated at 37°C for 3 h as described in previous studies. Finally, after removing the medium, it was washed with phosphate‐buffered saline (PBS) and 0.2 ml of dimethyl sulfoxide (DMSO), the plate shaken, and the absorbance was read at 550 nm (Sarika & James, 2016).

#### 2.2.12 Coating process

##### Coating treatments on shrimp

The shrimps were washed in cold water (<10℃) and were divided into four groups, including (1) control, (2) coated with PAGE, (3) coated with PAGE + curcumin (with curcumin concentration of 5 mg/ml), and (4) coated with curcumin‐loaded PAGE NPs (with curcumin concentration of 5 mg/ml). The shrimps were immersed in the respective coating solutions at 25℃ for 2 min. The control group was dipped into distilled water. Finally, the samples were exposed to a static airflow for 5 min and were packed in polyethylene pouches and kept at 4℃.

##### pH measurement

Ten grams of the sample was homogenized by using an ultratorax homogenizer (Janke and Kunkel, Germany) at 24,000 *g* in 90 ml double‐distilled water, and pH of the supernatant was recorded by a digital pH meter.

##### Total volatile basic nitrogen (TVB‐N)

To measure TVB‐N of the samples, 10 g of the crushed sample was mixed with 100 ml of water, followed by homogenizing and filtering. Afterward, 5 ml of the filtrate and 5 ml MgO solution were added to the reaction chamber. 10 ml Boric acid and methyl red and methylene blue (with the respective ratio of 2:1) was also added to the reaction chamber. Finally, the resulting solution was titrated with 0.1 mol/L HCl solution. The amount of TVB‐N was reported as mg nitrogen (mg/100 g).

### 2.3 Statistical analysis

The data were reported as mean ± *SD*. The significance of differences between the average values of results was analyzed by analysis of variance (*p* ≤ .05) with Duncan's test with SPSS software version 16 (IBM software, NY, USA). Curve fitting was carried out by DD‐SOLVER.

## 3 RESULTS AND DISCUSSION

### 3.1 Nanoparticles characterization

#### 3.1.1 Particle diameter and polydispersity index

The PAGE nanoparticles were fabricated by electrostatic interaction between Ca^2+^ ions and carbonyl/carboxylic acid functional groups on PAGE macromolecular chains. Preliminary experiments demonstrated that the pH of the medium and CaCl_2_ concentration profoundly affected the particle diameter, polydispersity index (PDI), surface charge, and morphological properties of nanoparticles. Therefore, the impact of these variables on particle diameter, surface charge, and PDI of the samples was investigated to find the optimal condition for the fabrication of nanoparticles.

According to the available literature, the particle adsorption correlates with the size, where the smaller size of particles, the more adsorption. From a pharmaceutical point of view, the submicron‐sized particles are favorable (Tiwari & Takhistov, 2012). Hence, nanoparticles formulation with the smallest size was chosen for the encapsulation of curcumin. To fabricate the nanoparticles with small size, the polymers solutions with a low concentration (in the dilute regime) should be used. In this region, due to the lower resistance of the liquid phase against dispersion, the particle size decreases (Mohammadpour Dounighi et al., 2012). Hence, in this study, PAGE solution with a concentration of 0.1% (w/w) (below critical point) was used to fabricate PAGE‐based particles.

As summarized in Table 1, following an increase in CaCl_2_ concentration, the diameter of nanoparticles increased. This trend is due to the increase of the average electrostatic interaction with the increase of ion concentration, which results in the production of larger particles (Koo et al., 2011). Likewise, previous researchers observed that increasing CaCl_2_ concentration led to the increase of the particle size (Koo et al., 2011; Liu & Gao, 2009; Naji‐Tabasi et al., 2017). On the other hand, Daemi and Barikani (2012) reported that higher ion concentration led to a more compact structure and smaller size.

**TABLE 1:** Particle diameter, size distribution, and surface charge of the nanoparticles in various pHs and CaCl_2_ concentration

Sample	pH	CaCl_2_ Conc	Particle size (nm)	PDI	Zeta Potential (mV)
1	6	1	652.9 ± 10.1^c^	0.33 ± 0.02^a^	−16.3 ± 2.6^e^
2	6	3	693.7 ± 14.0^b^	0.32 ± 0.03^a^	−12.7 ± 4.1^f^
3	6	5	820.3 ± 12.9^a^	0.32 ± 0.04^a^	−9.1 ± 2.3^g^
4	7	1	193.1 ± 18.0^f^	0.29 ± 0.06^a^	−39.3 ± 4.1^c^
5	7	3	220.1 ± 10.2^ef^	0.26 ± 0.02^ab^	−36.2 ± 2.1^c^
6	7	5	269.8 ± 6.8^d^	0.27 ± 0.04^ab^	−22.6 ± 1.8^d^
7	8	1	126.1 ± 4.5^h^	0.22 ± 0.07^b^	−56.8 ± 3.3^a^
8	8	3	163.0 ± 8.12^g^	0.22 ± 0.06^b^	−44.2 ± 4.3^b^
9	8	5	192.3 ± 13.7^f^	0.24 ± 0.08^b^	−35.5 ± 3.1^c^

Note

a, b, c, d, e, f, and g, different letters in the same column indicate significant differences at 5%.

The particle diameter of the nanoparticles was determined in three pH levels of 6, 7, and 8 (Table 1). The particle size of the samples which were fabricated at pH 8 was smaller than those of pH 6 and 7. In pH 8, the gum has a greater surface charge and more ability to interact with Ca^2+^ ions to form the smallest particles. On the other hand, around acid dissociation constant of PAGE (≈3.3), the electrostatic repulsive forces decreased, and flocculation occurred, and as a result, the particle diameter increased. The same observations have been reported by Maldonado et al. (2017), who examined the effect of pH on the nanoparticle characteristics of bovine serum albumin and poly‐d‐lysin. The smallest diameter of nanocapsules was observed for sample 7 (pH 8 and CaCl_2_ concentration of 1%).

The influence of pH and ion concentration on PDI value of nanoparticles is given in Table 1. A lower value of PDI shows a narrow unimodal particle size distribution. PDI values lower than 0.3 indicate a homogeneous dispersion, whereas those higher than 0.3 show a high level of heterogeneity (Hasan et al., 2016). The PDI value of all the samples fabricated in pH 7 and 8 (except for sample 4) is lower than 0.3, demonstrating a high level of homogeneity. The increase of CaCl_2_ concentration had no significant effect on PDI value of nanoparticle dispersion, which is consistent with those observed by Naji‐Tabasi et al. (2017). On the other hand, with an increase of pH value, solubility and ionization degree of PAGE increased which induced the homogeneity in particle size distribution. The same observation has been reported by Tan et al. (2016).

#### 3.1.2 Surface charge of nanoparticles

The surface charge of nanoparticles is one of the most critical factors to fabricate small and uniform particles with high stability. For a dispersion containing nanoparticles with high surface charge, the particles repel each other because of a repulsive force, making the suspension stable (Rydström Lundin, 2012). The values of zeta potential of nanoparticles are presented in Table 1. All the developed nanoparticles showed a negative charge (−9.1 to −56.8 mV) which is due to the anionic nature of PAGE. As shown in Table 1, the absolute value of surface charge for five formulations (4, 5, 7, 8, and 9) was higher than 30 mV. Such high zeta potential made the suspension stable. As expected, as the CaCl_2_ concentration elevated, the absolute value of the zeta potential of nanoparticles declined. This decreasing trend is probably due to the naturalization of acidic groups in PAGE structure with Ca^2+^ ions. At acidic condition, the surface charge of the samples was lower than those of natural and alkaline medium. This observation could be associated with the carboxylic acid protonation of the nanoparticles around the acid dissociation constant of PAGE, which led to a reduction of surface charge (Lv et al., 2019). In conclusion, the results demonstrated that formulation 7 (pH 8 and CaCl_2_ concentration 1%) resulted in the smallest particle size and PDI and highest surface charge, and thus, it is suitable for the encapsulation of curcumin. Other tests were carried out on the nanoparticles fabricated in optimal condition (sample 7).

### 3.2 Efficiency and yield of encapsulation (EE)

EE is an indicator for evaluating performance of encapsulation process. The EE in the nanoparticles was 86.1%, demonstrating that the encapsulation method applied in the current study was effective to entrap most of the curcumin within the PAGE matrix. However, it should be noted that 13.9% of curcumin could be distributed on the surface of the nanoparticles and could be extracted using a hydrophobic solvent. In general, the high value of EE supported the viability of the developed encapsulation system for the retention of curcumin.

### 3.3 Morphological properties

**FIGURE 1:**
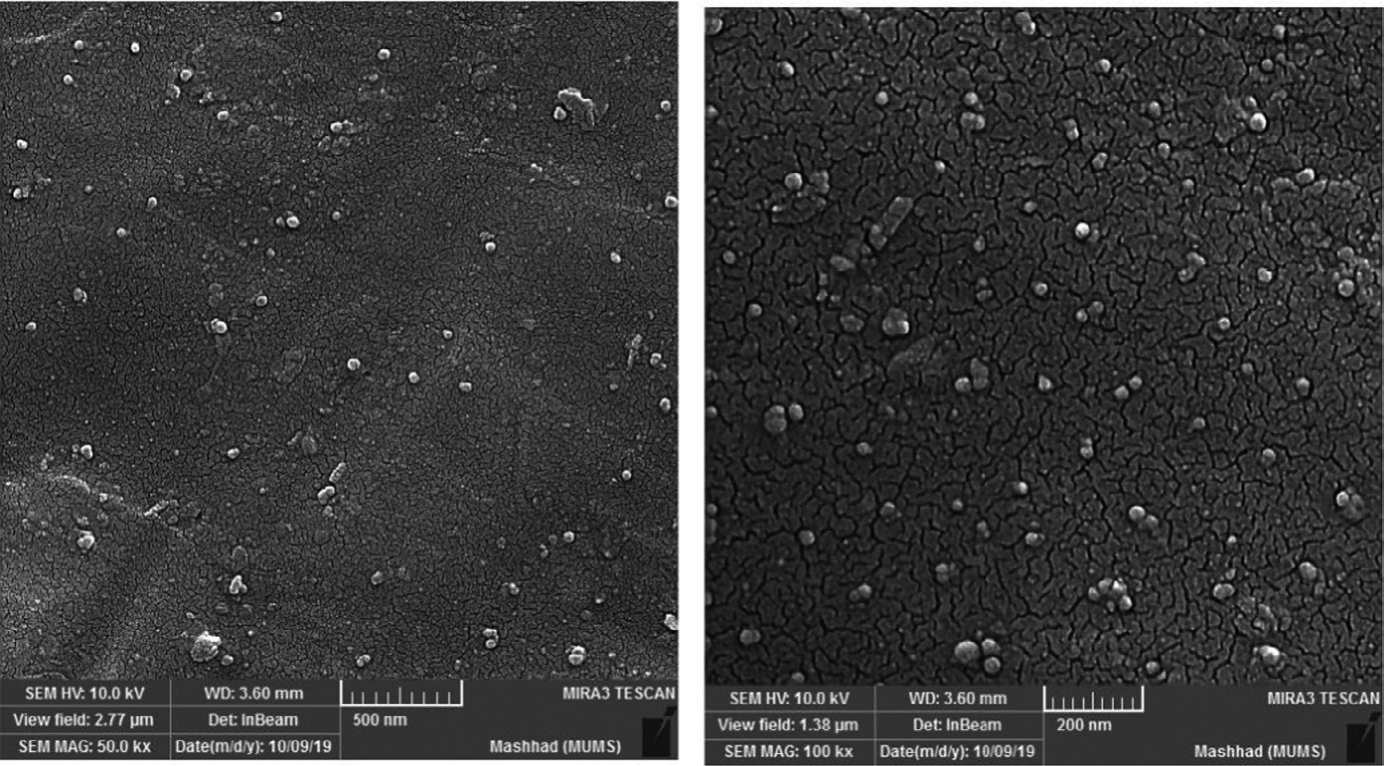
SEM images of PAGE nanoparticles containing curcumin

The morphological properties of nanoparticles have a considerable impact on the release profile of encapsulated compounds (Fathi et al., 2021). SEM analysis was used to evaluate the microscopical properties of the developed nanoparticles. SEM image demonstrated that the nanoparticles were smooth‐surface spherical in shape and had a size range of 50–100 nm (Figure 1). According to the literature, the particle diameter has an important role in the accumulation of particles in tumor cells. Tumor vasculature cutoff is in the range of 200–800 nm, and hence, the particles with small size can easily penetrate into the tumor cells (Sarika et al., 2015).

The surface roughness of nanoparticles is a determinant factor in determining the cellular uptake pathway and amount [30]. For instance, it is proved that the cellular uptake of smooth NPs in HeLa cells, as a cell line model, was faster than the uptake of rough nanoparticles. Hence, it is expectable that the developed nanoparticles can easily be taken up by cells. The appearance of nanoparticle observed in the present work is consistent with that reported by Noronha et al. (2013), where Poly ɛ‐caprolactone was used as wall material to encapsulate curcumin using nanoprecipitation method. The nanoparticles displayed smaller size by using SEM as compared to nanoparticles observed by previously described DLS analysis. This difference is because of the inherent variance in the measurement of particle diameter between SEM and DLS. In DLS method, the size is measured in dispersion, whereas SEM reported the diameter of particles in lyophilized samples.

### 3.4 FT‐IR and XRD analysis

**FIGURE 2:**
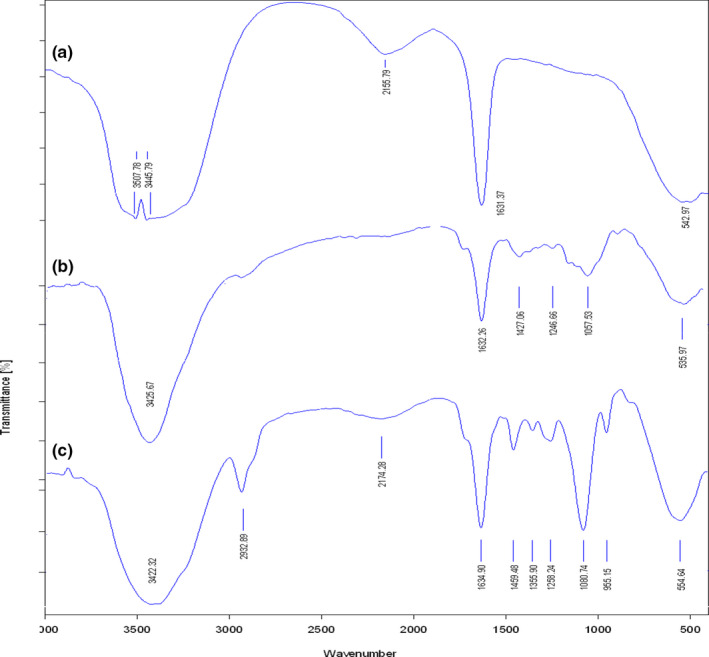
FT‐IR spectra of CaCl_2_ (a), PAGE (b), and PAGE nanoparticles (c)

FT‐IR is commonly used as an informative tool to investigate the structural characteristics of nanoparticles. The FT‐IR spectra of PAGE, CaCl_2,_ and nanoparticles are shown in Figure 2. In PAGE spectrum, the wavenumber at 1057.53 cm^−1^ is assigned to O‐acetyl groups (Percival, 1962). The peak located at 1427.06 cm^−1^ is due to the symmetrical stretching of carboxylate groups in uronic acid structure (Vinod et al., 2008) and the main one at 1632.26 cm^−1^ is due to asymmetric stretching C‐OO groups, which confirmed the presence of uronic acid in PAGE structure. In the spectrum of nanoparticles, the intensity of the peaks related to symmetrical and asymmetric stretching of carboxylate groups changed greatly. The intensity of these absorptions became stronger in PAGE nanoparticles than PAGE, because of the combination of the peaks at the 1631.37 and 1632.26 cm^−1^ for CaCl_2_ and PAGE, respectively. The combined peak was indicated a right‐shifting to 1634.90 cm^−1^. This observation confirmed the existence of electrostatic interaction between Ca^2+^ and COO‐ of PAGE. These observations are in agreement with those reported by Tan et al. (2016), who fabricated chitosan‐Arabic gum nanoparticles.

**FIGURE 3:**
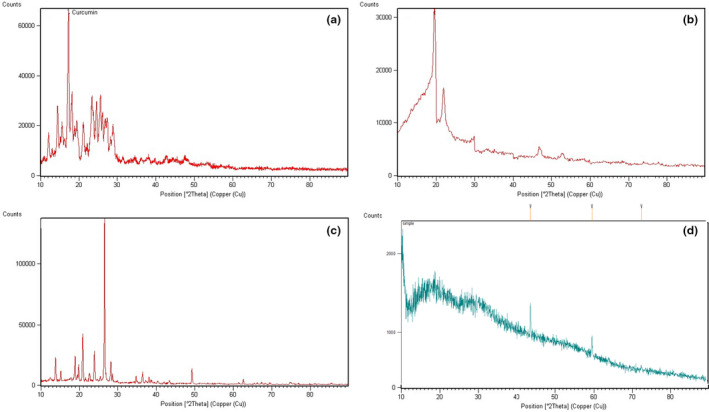
XRD patterns of curcumin (a), PAGE (b), CaCl_2_ (c), and curcumin‐loaded nanoparticles

XRD evaluation is applied to evaluate the crystallinity degree of components. Here, XRD was used to analyze the crystal transformation of developed nanoparticles. The XRD patterns of PAGE, CaCl_2,_ and PAGE nanoparticles are shown in Figure 3. There were several crystalline peaks in CaCl_2_ diffraction. A peak was also detected at 23° in PAGE. Nevertheless, the crystalline peaks disappeared in the XRD pattern of nanoparticles, demonstrating the disruption of the crystallinity of PAGE after electrostatic interaction with CaCl_2_. These observations are in agreement with those obtained in the FT‐IR test described above.

### 3.5 Release studies

**FIGURE 4:**
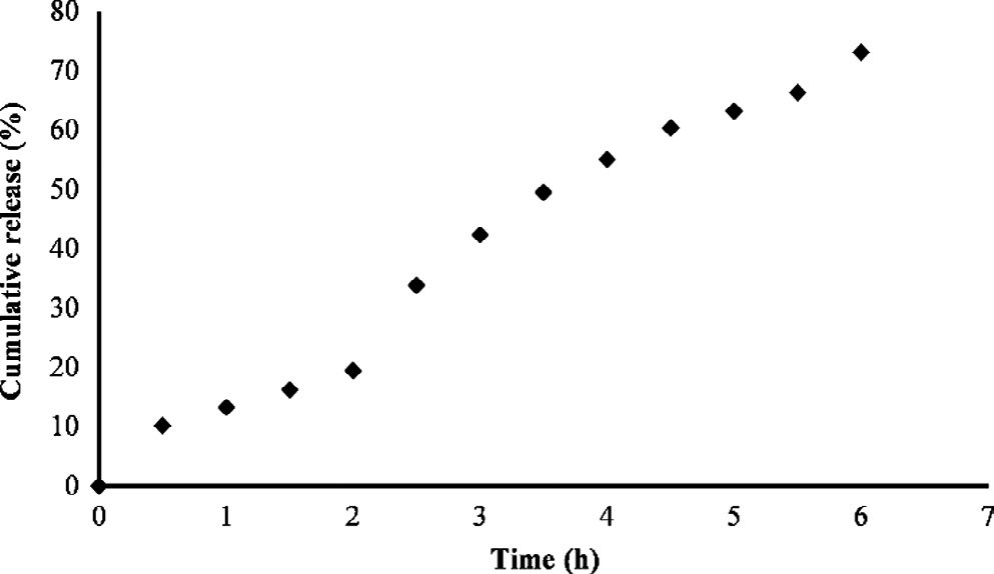
Cumulative release profile of curcumin from PAGE nanoparticles at simulated gastrointestinal tract

The cumulative release profile of curcumin from PAGE nanoparticles at simulated gastrointestinal tract is depicted in Figure 4. Only 19.1% of curcumin was released from fabricated nanoparticles in simulated stomach conditions. This observation may be attributed to a strong matrix polysaccharide network that suppressed the release of curcumin. Furthermore, the change in the pH and the ionic strength can lead to swelling and shrinking of polysaccharides. This property can be used to trigger the release of a bioactive compound (Jelvehgari et al. 2014, Sonia & Sharma, 2011). For instance, anionic polysaccharide‐based nanoparticles shrink in acidic medium. The shrinkage reduces the release rate of curcumin from nanoparticles (Tan et al., 2016). For the oral delivery purpose, this slow release of curcumin in the stomach is suitable because the higher amount of bioactive compound is available for intestine adsorption (Tan et al., 2016).

Cumulative release of curcumin in the gastrointestinal condition was found to be <75%, indicating a gradual release trend. The sustained release of curcumin from PAGE nanoparticles improves the bio‐accessibility of curcumin. The penetration of bile salts and pancreatin led to the leakage of curcumin (Andrieux et al., 2009). PAGE‐based nanoparticles can be introduced as an appropriate vehicle to sustain and control release of curcumin for a long duration.

Table 2 presents the correlation coefficient (*R* ^2^), Adj‐*R* ^2,^ and root mean square error (RMSE) of the release profile at tested kinetic models. It can be observed that Peppas model had the highest *R* ^2^ and adj‐*R* ^2^ and lowest RMSE. *n* parameter in Peppas model shows the drug transport mechanism, which is dependent on the geometry of particles (Siepmann & Peppas, 2012). According to SEM analysis, the PAGE nanoparticles had spherical shape as represented in Figure 1a, b. According to Siepmann and Peppas (2012), for nanoparticles with spherical shape, a value lower than 0.43 demonstrates Fickian diffusion, while a value between 0.44 to 0.85 presents Anomalous transport. A value higher than 0.85 shows Case‐II transport. The value of *n* for PAGE nanoparticles was found to be 0.94, which demonstrates the curcumin release obeyed the Case‐II transport mechanism.

**TABLE 2:** Modeling of release profile of curcumin from PAGE nanoparticles in the simulated gastrointestinal tract

Model	*R* ^2^	Adj‐*R* ^2^	RMSE	k	*n*
Zero model	0.97	0.97	3.50	12.78	–
First‐order model	0.96	0.96	4.98	0.187	–
Higuchi model	0.86	0.86	9.19	25.65	–
Peppas model	0.99	0.98	3.6	13.94	0.94

### 3.6 Antimicrobial activity

In recent years, several studies have been focused on the examination of the antimicrobial capacity of free and encapsulated curcumin against pathogenic microorganisms. In this study, the antimicrobial capacity of bare and curcumin‐loaded PAGE NPs against *Staphylococcus aureus* and *Escherichia Coli* was examined. Both free and encapsulated curcumin showed a clear inhibition zone. The antimicrobial activity of curcumin has been associated with the hydrogen‐bonding and hydrophobic interactions of this phenolic compound with membranal proteins of the bacterial cells that change the permeability of the membrane, and as a result, inhibit the bacterial growth (Aliabbasi et al., 2021). Wang et al. (2017) also indicated that the antibacterial activity of curcumin may be due to the changes occurred in the shape of bacterial cells in the presence of curcumin. Comparatively, the antimicrobial capacity of curcumin‐loaded PAGE nanoparticles was more when compared with free curcumin. This observation has been attributed to the smaller size of curcumin‐loaded nanoparticles than curcumin particles dissolved in DMSO, (500–800 nm), which facilitates the penetration of curcumin into the bacteria cells (Bhawana et al., 2011).

The antibacterial activity of PAGE‐based nanoparticles without curcumin was also investigated. The results demonstrated that PAGE nanoparticles had no antimicrobial activity against tested bacteria.

From Table 3, the antimicrobial activity of free and encapsulated curcumin against Gram‐positive bacteria was more pronounced than that of Gram‐negative bacteria. This is because of two peptidoglycan layer present in the cell's structure of gram‐negative bacteria that protect them against many antimicrobial agents like curcumin (Salarbashi et al., 2018).

**TABLE 3:** Inhibition zone of free and encapsulated curcumin

	Zone inhibition (mm)	
	Organism	
	*Staphylococcus aureus*	*Escherichia Coli*
Curcumin (DMSO)	5.2 ± 0.2^b^	3.1 ± 0.2^b^
Curcumin‐loaded PAGE NPs	7.0 ± 0.4^a^	3.9 ± 0.1^a^
PAGE NPs without curcumin	–	–

Note

a and b, different letters in the same column indicate significant differences at 5%.

### 3.7 Cytotoxicity studies

**FIGURE 5:**
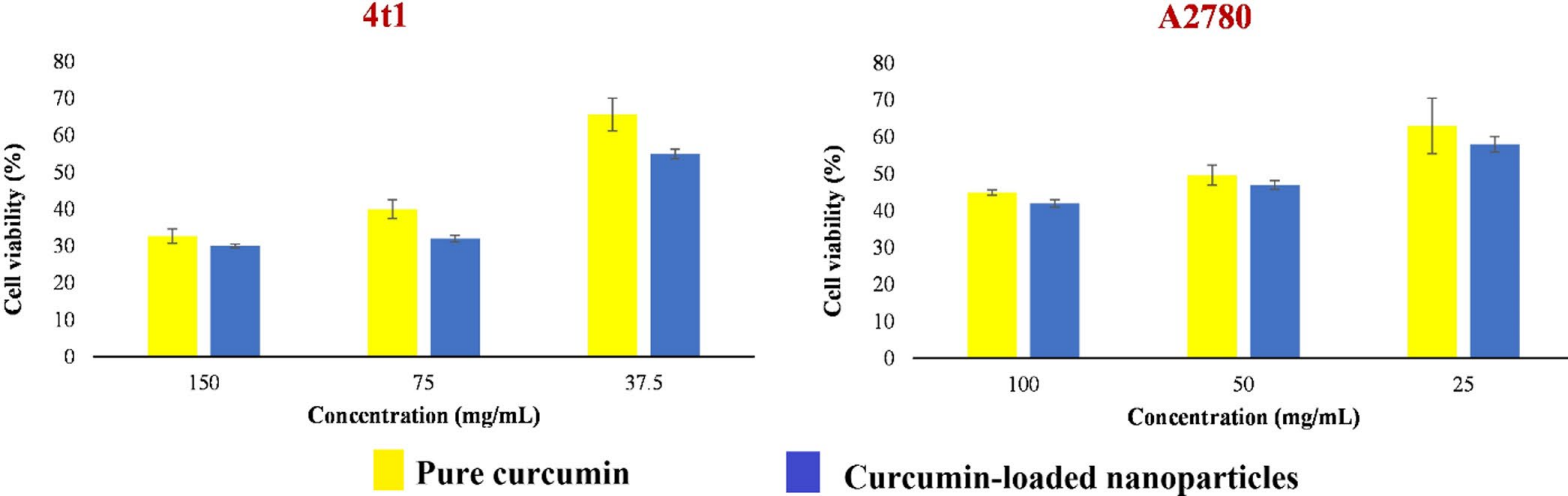
Anticancer activity of pure and encapsulated curcumin against two carcinogenic cell lines including 4t1 and A2780

The cytotoxicity of encapsulated curcumin against two cell lines including 4t1 and A2780 was compared to pure curcumin by MTT test to evaluate whether the curcumin‐loaded nanoparticles can provide cytotoxicity or not. As shown in Figure 5, both pure curcumin and curcumin‐loaded nanoparticles were toxic to the tested cell lines. It also can be observed that the cytotoxicity of the tested samples was concentration‐dependent. The curcumin‐loaded nanoparticles showed stronger cytotoxic effect, demonstrating synergistic effect of curcumin and PAGE.

### 3.8 The application of PAGE, PAGE + curcumin, and curcumin‐loaded PAGE nanoparticles as coating for shrimp

Generally, determining TVB‐N content and pH are two common chemical methods for inspecting meat quality (Kuswandi & Nurfawaidi, 2017).

#### 3.8.1 pH value

**FIGURE 6:**
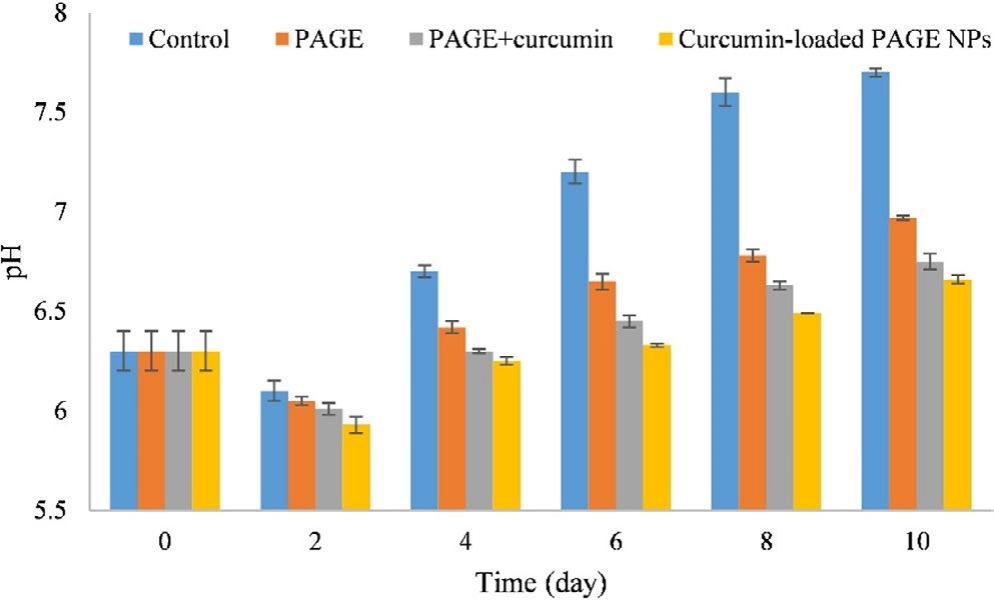
Changes in pH values of fresh and coated shrimp over storage time

Change in pH values is commonly considered as a measure for evaluating the freshness of shrimp. The changes in the pH of fresh shrimps (control), coated with PAGE, coated with PAGE + curcumin, and coated with curcumin‐loaded PAGE NPs throughout storage are presented in Figure 6. It can be observed that the pH of control sample was higher than those recorded for the coated shrimps. With increasing storage time up to a certain point, the pH of all samples declined and beyond that increased gradually. This observation is consistent with those reported by Mu et al. (2012) and Zhang et al. (2019). The initial decrease in pH value of shrimp has been attributed to the production of acidic components, such as lactic acid and phosphoric acid over storage period of shrimps (Qiu et al., 2014). The pH elevation in later stage is caused by the presence of volatile amines that are formed by the decomposition of the proteins and microbial activity.

The lowest pH value (6.66) after 10 days was obtained in the shrimps coated with curcumin‐loaded PAGE NPs. The pH values of the samples coated with PAGE and PAGE+curcumin were 6.97 and 6.75, respectively, which were lower than the pH value of control sample (7.7) (*p* ˂ .05). More efficiency of curcumin‐loaded PAGE NPs than PAGE+curcumin for maintaining quality of shrimp and increasing its shelf life may be related to the sustained release of curcumin as demonstrated in the in vitro curcumin release profile. Overall, curcumin‐loaded PAGE NPs can be introduced as promising coating agents to preserve the quality of shrimp and reduce decomposition rates of shrimp during storage period.

#### 3.8.2 TVP‐N content

**FIGURE 7:**
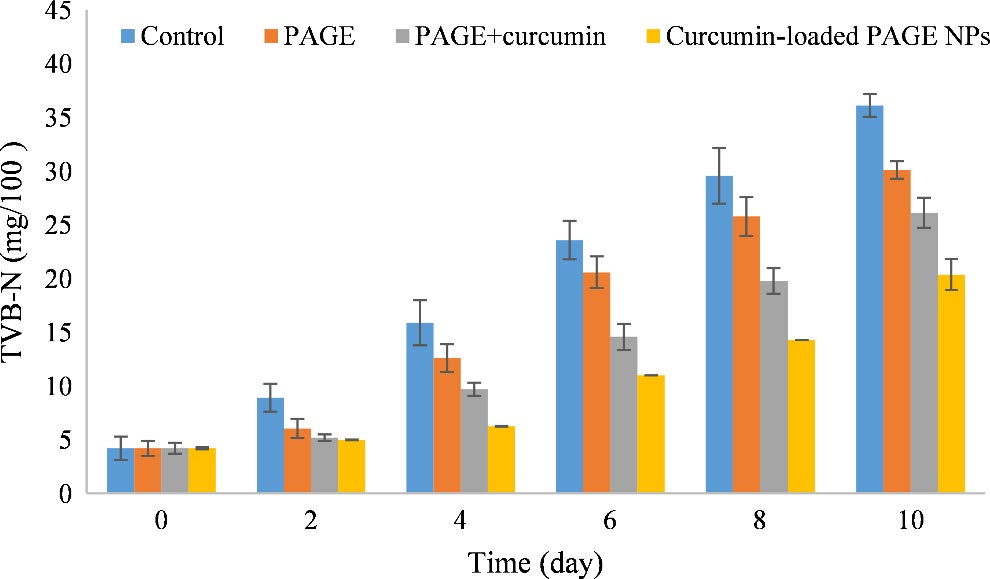
Changes in TVB‐N content of fresh and coated shrimp over storage time

During storage of seafoods, various nitrogenous compounds, such as TVB‐N, are formed that change the pH value of these foodstuff. As shown in Figure 7, when the storage time increased from 0 to 10 days, the TVB‐N contents of control sample continuously increased from 4.2 ± 2.14 mg/100 g to 36.14 ± 1.06 mg/100 g, which was significantly higher (*p* < .05) than those observed for the coated samples. TVB‐N contents for the samples coated with PAGE, PAGE + curcumin, and curcumin‐loaded PAGE NPs were found to be 30.14 ± 0.83 mg/100 g, 26.15 ± 1.39 mg/100 g, and 20.39 ± 1.44 mg/100 g, respectively, at 10th day. Based on the hygienic standards for marine products, the rejection limit of TVB‐N content for shrimp is more than 30 mg/100 g. Therefore, the coated shrimps were in fresh period during storage time. The reason for the lowest TVB‐N contents in the curcumin‐loaded PAGE NPs group is attributed to antioxidative and antibacterial activity of curcumin as well as the sustained release of curcumin.

## 4 CONCLUSION

Based on the proposed aims and the results obtained, this work demonstrated that the use of PAGE nanoparticles provided the small particle sizes with high level of homogeneity and surface charge. The shape of fabricated nanoparticles was spherical with smooth surface. The formation of complex coacervation was confirmed by FT‐IR and XRD studies. The gradual release of curcumin from PAGE nanoparticles in gastrointestinal tract was observed. Thus, this delivery system can be used for food and pharmaceutical applications. The release profile of curcumin followed Peppas model in gastrointestinal medium with *n* value of 0.94, indicating the curcumin release obeyed Case‐II transport mechanism. Both pure curcumin and curcumin‐loaded nanoparticles were toxic to the cancer cell lines, demonstrating the encapsulation of curcumin into the developed system had no negative effect on its anticancer activity. In the present study, cucrcumin‐loaded PAGE NPs presented enhanced preservation performance on shrimp than PAGE alone or PAGE combined with curcumin.

## AUTHOR CONTRIBUTION

**Davoud Salarbashi:** Conceptualization (equal); Formal analysis (lead); Project administration (equal). **Mohsen Tafaghodi:** Conceptualization (equal); Investigation (equal); Validation (equal); Visualization (equal). **Morteza fathi:** Investigation (equal); Software (equal); Writing‐original draft (lead). **Seyyed Mohammad aboutorabzade:** Investigation (equal); Software (equal); Writing‐review & editing (equal). **Farzaneh Sabbagh:** Investigation (equal); Writing‐review & editing (equal).
